# Cellulose Acetate 398-10 Asymmetric Membrane Capsules for Osmotically Regulated Delivery of Acyclovir

**DOI:** 10.1155/2016/8471520

**Published:** 2016-02-11

**Authors:** Alka Sonkar, Anil Kumar, Kamla Pathak

**Affiliations:** Department of Pharmaceutics, Rajiv Academy for Pharmacy, Mathura, Uttar Pradesh 281001, India

## Abstract

The study was aimed at developing cellulose acetate asymmetric membrane capsules (AMCs) of acyclovir for its controlled delivery at the absorption site. The AMCs were prepared by phase inversion technique using wet process. A 2^3^ full factorial design assessed the effect of independent variables (level(s) of polymer, pore former, and osmogen) on the cumulative drug release from AMCs. The buoyant optimized formulation F7 (low level of cellulose acetate; high levels of both glycerol and sodium lauryl sulphate) displayed maximum drug release of 97.88 ± 0.77% in 8 h that was independent of variation in agitational intensity and intentional defect on the cellulose acetate AMC. The* in vitro* data best fitted zero-order kinetics (*r* ^2^ = 0.9898). SEM micrograph of the transverse section confirmed the asymmetric nature of the cellulose acetate capsular membrane. Statistical analysis by Design Expert software indicated no interaction between the independent variables confirming the efficiency of the design in estimating the effects of variables on drug release. The optimized formulation F7 (desirability = 0.871) displayed sustenance of drug release over the drug packed in AMC in pure state proving the superiority of osmotically active formulation. Conclusively the AMCs have potential for controlled release of acyclovir at its absorption site.

## 1. Introduction

Asymmetric membrane capsule is a controlled drug delivery device which consists of a drug core surrounded by a membrane of asymmetric structure (relatively thin, dense region supported on a thicker, porous region). Similar to a conventional hard gelatin capsule, the asymmetric membrane capsule (AMC) consists of a cap and a body that snugly fit into each other. The cap is shorter in length and has a slightly larger diameter than the body which is longer and has a smaller diameter. In contrast to gelatin capsules, however, the walls of AMCs are made from water-insoluble polymer(s) such as cellulose acetate, ethyl cellulose, cellulose acetate butyrate, and their mixtures [1]. Thus, the capsule shell does not dissolve to instantly release the drug filled in it. Instead, the drug is released over a prolonged duration by diffusion through the capsule walls and/or via osmotic pumping by convection through pores in the capsule walls [2]. The use of asymmetric membranes as rate controlling membrane of drug delivery devices is being widely explored. The basic mechanism of drug release from asymmetric membrane capsule is osmosis. When the capsule comes into contact with water, water imbibes into it and dissolves the soluble component in the core, forming the solution of the drug. The hydrostatic pressure was generated within the core which acts as a driving force to deliver the drug through preexisting pores, after all components are depleted and asymmetric membrane coating remains intact [3].

Asymmetric membrane capsules have been proven to be efficient gastroretentive systems carriers for osmotically regulated delivery of highly water-soluble drug, ranitidine hydrochloride, by a report published from our lab [4]. This concept is being extrapolated for a poorly water-soluble drug, acyclovir, based on the literature support of suitability of AMCs for delivery of poor water-soluble drug due to high water flux capability [5–7].

Acyclovir is an antiviral agent used for the treatment of* Herpes simplex* virus types I and II and* Varicella zoster* virus. It has an oral bioavailability of 10–20% with a very short biological half-life of 2–4 h, so high frequent dosing is required [8]. The absorption of acyclovir from the gastrointestinal tract is variable and incomplete; 10–30% of an oral dose may be absorbed [9]. Because of its high hydrophilic nature, absorption of acyclovir occurs mainly by passive diffusion mechanism and is slow, variable, and incomplete [10]. Food does not appear to affect gastrointestinal absorption [11]. The absorption window of the drug is in the stomach and upper part of the intestine that can result in incomplete drug release from the drug delivery system leading to reduced efficacy of the administered dose. Hence designing a gastroretentive formulation that would provide controlled release of the drug may offer reduction in total dose and frequency of administration and enhanced absorption and hence bioavailability.

Thus, the aim of the project was to optimize cellulose acetate AMCs for osmotically controlled gastroretentive delivery of acyclovir using 2^3^ factorial design. Acyclovir was selected as an active agent as it met the desired criteria for being the potential candidate for asymmetric membrane technology controlled drug delivery system: (i) poor aqueous solubility (2.5 mg/mL), (ii) short plasma half-life (2–4 h), and (iii) absorption that is unaffected by presence of food in stomach. Prior to developing AMCs, the solubility of acyclovir was modulated for achieving a controlled release formulation.

## 2. Materials and Methods

### 2.1. Materials

Acyclovir was a kind gift from Zen Lab & Preet Remedies Pvt. Ltd., Baddi, India.

Cellulose acetate 398-10 was brought from Sigma Aldrich Chemical, Germany; glycerol was brought from S. D. Fine Chemicals, Mumbai, India; sodium lauryl sulphate was brought from Ranbaxy Fine Chemicals Ltd., New Delhi, India. Acetone, ethanol 95% v/v, potassium dihydrogen orthophosphate, and methylene blue were obtained from S. D. Fine Chemicals, Mumbai, India.

### 2.2. Equilibrium Solubility and Its Modulation

An excess amount of acyclovir was suspended in 10 mL each of double distilled water and phosphate buffer, pH 4.5, and maintained at 37 ± 0.5°C. The flasks were then shaken for 72 h in water bath shaker (Hicon Enterprises, New Delhi, India). The suspension was filtered through 0.2 *μ*m size filter paper and the temperature was maintained at 37 ± 0.5°C using lab fabricated temperature regulating boxes. The solution was analyzed spectrophotometrically at 252 nm in a double-beam UV spectrophotometer (Shimadzu Pharmaspec-1700, Kyoto, Japan) after appropriate dilution. The solubility was determined using validated calibration curve. The solubility of acyclovir was modulated by sodium lauryl sulphate. To solutions of sodium lauryl sulphate of varying molar strength (0.25, 0.50, 0.75, 1.00, 1.25, 1.50, and 1.75 M) in double distilled water and in phosphate buffer, pH 4.5, excess drug was added and solubility determination was carried out as described in the preceding text.

### 2.3. Differential Scanning Calorimetry

The differential scanning thermograms profiles of pure drug, excipients, and physical mixtures thereof were recorded on DSC-60 controlled by TA-60 WS software (Shimadzu, Kyoto, Japan). The samples were weighed and transferred to the equipment for analysis in hermetically sealed aluminium pans. An indium standard was used to calibrate the differential scanning calorimeter temperature. The samples were heated, over a temperature range of 0–300°C. An inert atmosphere was maintained by purging with nitrogen at the flow rate of 20 mL/min.

### 2.4. Determination of Controlled Release Dose

The controlled release dose was calculated using the following equation [12]:

(1) $$\begin{array}{c} D_{t}=D_{i}\left(\;1+0.693\times \frac{t}{t_{1/2}}\;\right), \end{array}$$

where *D*_*t*_ is total dose required for the dosage form, *t* is time for drug release (8 h), *t*_1/2_ is half-life of the drug (4 h), and *D*_*i*_ is immediate release dose (200 mg). Thus *D*_*t*_ was calculated as 477.2 mg and for the experimental purpose it was rounded off to 480 mg.

### 2.5. Experimental Design

A 2^3^ full factorial design [13] was utilized for the formulation and optimization of AMCs. The independent variables in the study were concentration of cellulose acetate 398-10 (A), concentration of glycerol (B), and content of sodium lauryl sulphate (C). For each of these variables, an experimental range in terms of levels was selected based on the results of preliminary experiments. Each factor was taken at two levels (+1, −1), which were coded as high or low levels, respectively. The response was percent cumulative drug release (% CDR) in 8 h. The composition of all the formulations (*n* = 8) with coded values was given (Table 1).

### 2.6. Fabrication of AMCs

Asymmetric membrane capsules were made by dip coating (phase inversion) process [14] wherein the polymeric membrane was precipitated on fabricated glass mould pins of diameters 7.30 ± 0.05 mm and 7.73 ± 0.02 mm for the body and the cap, respectively. The glass mould pins were dipped into polymeric solution of cellulose acetate 398-10 in acetone (50% v/v) and mixed with a mixture of glycerol in ethanol (25% v/v). The polymeric membrane precipitated on the pins was air-dried for 15 s, followed by dipping in aqueous quenching solution of glycerol for 10 min. Thereafter the pins were withdrawn manually and allowed to air-dry for at least 8 h. The capsules were stripped off the pins, trimmed to size, and kept in desiccators until use. The AMC's body was manually filled with a constant drug load mixed with sodium lauryl sulphate in accordance with the design. The body of the AMCs was then capped and sealed with 10% w/v sealing solution of cellulose acetate 398-10 in a mixture of acetone and ethanol (1 : 1).

### 2.7. Physical Characterization

The AMCs were characterized for surface, appearance, and dimensions and compared visually for transparency and opacity. Dimensions of AMCs were determined using vernier calliper. The results were statistically compared with conventional hard gelatin capsule of “zero” size at *p* < 0.05. A multiple of three determinants was used for measurement of each dimension.

### 2.8. Evaluation

#### 2.8.1. Uniformity of Weight

The weight of the capsule content was measured as a difference between the weight of intact capsule and that of the shell after removing the contents of the capsule. A total of twenty capsules were used for performing test and compared with the limit mentioned in Indian Pharmacopoeia, 2007 [15].

#### 2.8.2. Content of Active Ingredient

The amount of active ingredient in each capsule was determined as per method mentioned in Indian Pharmacopoeia, 2007. Five capsules from each formulation were used for the study.

#### 2.8.3. *In Vitro* Release

*In vitro* drug release test of the formulations was performed using USP paddle type II apparatus (Hicon Enterprises, New Delhi, India). The* in vitro* drug release was assessed in 900 mL of phosphate buffer, pH 4.5, stirred at 75 rpm, and maintained at a temperature of 37 ± 0.5°C for 8 h. Five milliliters of sample was withdrawn on hourly basis and the release medium was replenished with fresh dissolution media. The samples were suitably diluted with fresh media and analyzed spectrophotometrically at 252 nm. The studies were conducted in triplicate and the drug released at each time point was calculated as mean ± SE and plotted against time. The release data was modeled for zero-order, first-order, Higuchi square root, and Hixson-Crowell models [12] using PCP Disso Version 2.08 software, Pune, India. The criterion for selecting the most appropriate model was chosen on the basis of maximum linearity of the data to fit with the model. Additionally, each AMC formulation was monitored for its floating ability during* in vitro* drug release study and data was recorded every hour.

### 2.9. Statistical Analysis

The effect of independent variables on the responses was analyzed using Design Expert software version 9 (Stat-Ease, Inc., Minneapolis, USA). The polynomial equation was generated after omitting the insignificant coefficients at 95% confidence level using Pareto chart. The values of effects of coefficients were interpreted with the help of bar graph of coefficients obtained between the Bonferroni line and *t*-limit line. The generated polynomial equation for response parameter was used for validation of design. The 3D response surface graphs were used to analyze the influence of different levels of the variables on the response parameter (%  CDR_8 h_).

### 2.10. Selection of Optimized Formulation and Validation of Experimental Design

The optimized formulation was selected on the basis of maximum %  CDR_8 h_. The extra design checkpoint formulation (F9) was prepared by taking mean value of two levels for all three factors. The predicted value determined using polynomial equation was compared with experimental value at 95% confidence interval (*p* < 0.05).

### 2.11. Scanning Electron Microscopy

The asymmetric membrane was sputter-coated for 5–10 min with gold using fine coat ion sputter and examined under scanning electron microscope Ultra* Plus*, Carl Zeiss, Germany. The samples examined include (i) both sides of asymmetric membrane before and after* in vitro* release test, (ii) membrane with intentional defect before and after the* in vitro* release test, and (iii) transverse section of the membrane. On completion of the* in vitro* release study, asymmetric membrane structures were air-dried at 45°C for 12 h and stored between sheets of wax paper in desiccator until use.

### 2.12. Effect of Variables on Drug Release

In order to study the effect of variables on the drug release, the* in vitro* release test of optimized formulation was conducted under varied conditions. The effect of agitational intensity was studied by varying the rotational speeds (50, 100, and 150 rpm) of paddle (USP-II apparatus). Another factor studied was effect of intentional defect on the release of acyclovir.

A defect was intentionally incurred in the AMC using a needle so that a hole of 2 mm dia was made and the defective optimized formulation was subjected to* in vitro* release test.

The presence/absence of osmogen and its concentration in the formulation plays a vital role in deciding the release of drug from the AMC. In order to demonstrate this, four different experimental systems were used. The study was carried out by dye test wherein methylene blue was selected as color producing agent. The release of dye from the AMC was monitored in variable molar environment created by sodium lauryl sulphate inside and outside the capsule. Various osmotic conditions used for the study are documented in Table 2. The experimental setup(s) were stationed on lab shelf and the release of methylene blue was observed visually and interpreted. The release of the dye was indicative of its osmotic expulsion from core of capsule. Additionally, the osmotically regulated release of the drug was monitored quantitatively. The optimized formulation was introduced in phosphate buffer, pH 4.5 (900 mL), and variation in osmotic pressure was accomplished by controlling the amount of SLS in the capsule and in the surrounding environment. Condition A represents F7 without osmogen inside and outside the capsule that represented zero osmotic gradient; condition B represents F7 containing 290 mg SLS inside the capsule and 0 mg outside the capsule for perfect osmotic gradient; condition C represents 290 mg SLS inside and 145 mg outside the capsule (hypoosmotic); condition D represents F7 containing 290 mg SLS inside and 430 mg in the media for hyperosmotic condition. Conditions C and D were intended to analyze the effect of increasing the osmotic pressure (28.46 and 115.28 mmHg, resp., for C and D) of the external media on drug release.

### 2.13. Stability

The optimized formulation was subjected to stability testing as per ICH Q1 A [16]. Formulation F7 was sealed in aluminium foil coated inside with polyethylene and kept in stability chamber maintained at 40 ± 2°C and 75 ± 5% RH for 3 months. The samples were analyzed for any deterioration in terms of any changes in physical parameters (texture and color), the percent drug content, and* in vitro* drug release. The sampling intervals were 0, 1, 2, and 3 months.

## 3. Results and Discussion

### 3.1. Equilibrium Solubility and Its Modulation

**Figure 1:**
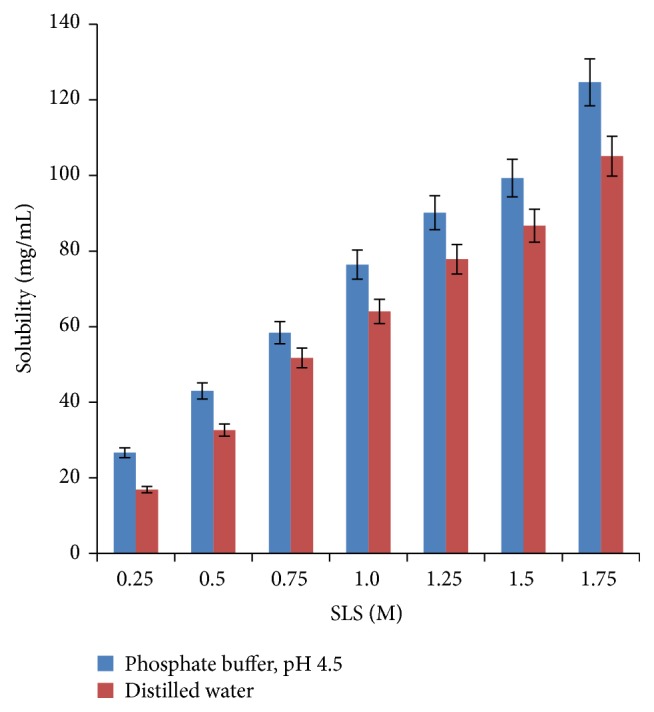
Bar chart for solubility modulation of acyclovir in the presence of sodium lauryl sulphate of varying molar strength.

The solubility studies data indicated that acyclovir was poorly soluble in both phosphate buffer, pH 4.5 (1.74 mg/mL), and double distilled water (1.36 mg/mL). The results are consistent with the literature report on acyclovir as “slightly soluble in water” at room temperature (22–25°C) and solubility values range from 1.2 to 1.6 mg/mL [17, 18]. The solubility is a prominent factor in governing the drug release from an osmotically controlled drug delivery system [19]. A poorly soluble drug (<10 mg/mL) will be governed by first-order release kinetics rather than zero-order kinetics. Hence, for achieving a controlled release formulation of acyclovir, its solubility was modulated using sodium lauryl sulphate. The target solubility range of 50–300 mg/mL [14] is presumed to be appropriate for controlled drug delivery from an osmotically regulated system. In the solubility modulation experiment, the solubility of acyclovir was observed to increase almost linearly with increasing molarity of sodium lauryl sulphate (Figure 1) in both phosphate buffer, pH 4.5, and distilled water.

The solubility enhancement is attributable to the micellization of acyclovir by sodium lauryl sulphate. The anionic with high HLB value of 40 [20] surfactant was selected as the osmogen owing to its high water solubility and it is GRAS listed and recommended to be employed in a wide range of nonparenteral pharmaceutical formulations. The solubility enhancement in phosphate buffer, pH 4.5, was of higher magnitude than in water (though not significant) at all molar strengths of sodium lauryl sulphate. At 0.25 M and 0.5 M strengths the solubility values were <50 mg/mL. A strength of 0.75 M of sodium lauryl sulphate resulted in solubility value(s) of 58.39 mg/mL in phosphate buffer, pH 4.5, and of 51.72 mg/mL in distilled water. Further, increase in strength to 1.0 M resulted in a solubility of 76.43 mg/mL in phosphate buffer, pH 4.5, and of 64.05 mg/mL in distilled water. Beyond this strength of SLS, the solubility increased but was not taken into consideration because it may not help in achieving sustained effect of the drug in the given dose of the drug.

### 3.2. Drug Excipient Compatibility

**Figure 2:**
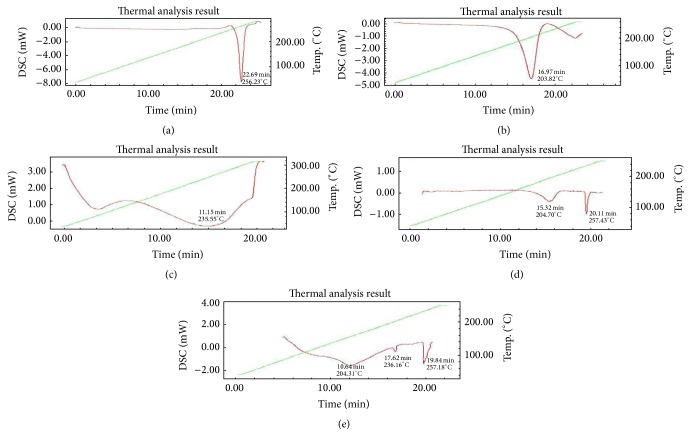
Differential scanning colorimetric thermographs of (a) acyclovir, (b) sodium lauryl sulphate, (c) cellulose acetate 398-10, (d) physical mixture of acyclovir and sodium lauryl sulphate, and (e) physical mixture of acyclovir, cellulose acetate 398-10, and sodium lauryl sulphate.

The physical mixtures of drug and excipients did not show any physical incompatibility in terms of caking, discoloration, odour, and liquefaction. Investigation of thermal behaviour of the stored samples resulted in differential scanning calorimetric profiles (Figure 2).

The thermogram of pure acyclovir revealed a sharp endothermic peak at 256.23°C (Figure 2(a)) representing the melting point in crystalline state [21]. At 203.82°C, a sharp endothermic peak was recorded for crystalline sodium lauryl sulphate (Figure 2(b)) and a broad endothermic peak at 235°C was observed for cellulose acetate 398-10 that showed broad peak (Figure 2(c)) corresponding to the amorphous nature of cellulose acetate [22]. The endothermic peak of the drug was retained in the physical mixture (Figure 2(d)) of acyclovir and sodium lauryl sulphate (257.43°C and 204.70°C) suggesting compatibility between the two. Furthermore, the peaks retained in the physical mixture of acyclovir with cellulose acetate 398-10 and sodium lauryl were retained at 257.18°C, 236.16°C, and 204.31°C, respectively (Figure 2(e)), with no significant shift. Absence of any peak confirmed the compatibility of the drug with the excipients.

### 3.3. AMCs of Acyclovir

#### 3.3.1. Appearance and Dimensions

The AMCs were opaque in appearance. The dimensions of the AMC capsule body and cap were significantly similar (*p* = 0.00116) to conventional hard gelatin capsules (Table 3). Furthermore, very low SE values around the mean suggest reproducibility of the method.

#### 3.3.2. Uniformity of Weight

The weight of AMCs varied between 697.60 ± 1.18 and 769.35 ± 0.74 mg (Table 4). The average weight of AMCs formulations was in accordance with IP guidelines [15]. Not more than two of the individual weights deviated from the average weight by more than 5% and none deviated by more than twice that percentage. This indicated uniform filling of powder blend in the capsule body.

#### 3.3.3. Content of Active Ingredient

The drug content of AMCs formulations was in accordance with IP guidelines. In all the eight formulations, the values for drug content closely ranged between 92.61 ± 1.44 and 99.45 ± 0.37% that ensured uniformity of the drug content in the capsules (Table 4).

#### 3.3.4. *In Vitro* Release

Among the formulations, the lowest drug release was observed from F2 (70.04 ± 1.36%) which may be due to the low level of glycerol (pore former) and sodium lauryl sulphate (osmogen) and high level of cellulose 398-10 (film former), since low level of glycerol leads to the formation of less porous film that did not provide the channels for water to get entered within the system and the low level of osmogen decreased the water influx and the high level of cellulose acetate 398-10 also provided thicker film that hinders the water to get penetrated and initiate the osmogen to exhibit osmotic effect [22]. F6 showed higher release (74.63 ± 0.46%) than F2 due to higher amount of osmogen, which led to increased water influx. F1 with lowest level of cellulose acetate 398-10 showed better release (78.07 ± 1.93%) than F2 and F6. This may be the result of formation of thinner asymmetric film of F1 that did not hinder the penetration of water and initiated the osmogen to exhibit its osmotic effect. F5 had better tendency to release the drug (80.59 ± 1.48%) due to higher amount of osmogen as compared to F1 and F2 since it had lower level of cellulose acetate as compared to F6. F4 exhibited higher drug release (84.11 ± 0.95%) than all the above-discussed formulations because the amount of glycerol was higher in F4 which facilitated the pore formation. F8 had higher amount of glycerol and higher amount of sodium lauryl sulphate thus giving further higher release of 86.86 ± 1.25%, due to the combined effect of increased pores and hence higher water influx. Similarly, highly porous film of F3 made of lower level of cellulose acetate and higher level of glycerol provided channels for water to enter the system and facilitate drug release to the tune of 91.16%. Finally, F7 made with high levels of both glycerol and sodium lauryl sulphate and low level of cellulose acetate 398-10 showed highest drug release of 97.88 ± 0.77%. The superior effect of F7 was a result of formation of a highly porous film that facilitated high water uptake and was selected as the optimized formulation. Most of the resistance to mass transfer is exerted by the dense portion of the membrane while the porous substrate provides mechanical strength and durability to the membrane [23]. The results of effect of formulation variables on drug release are consistent with our previous reports on osmotically regulated systems of poorly water-soluble drugs from AMCs [24, 25].

**Figure 3:**
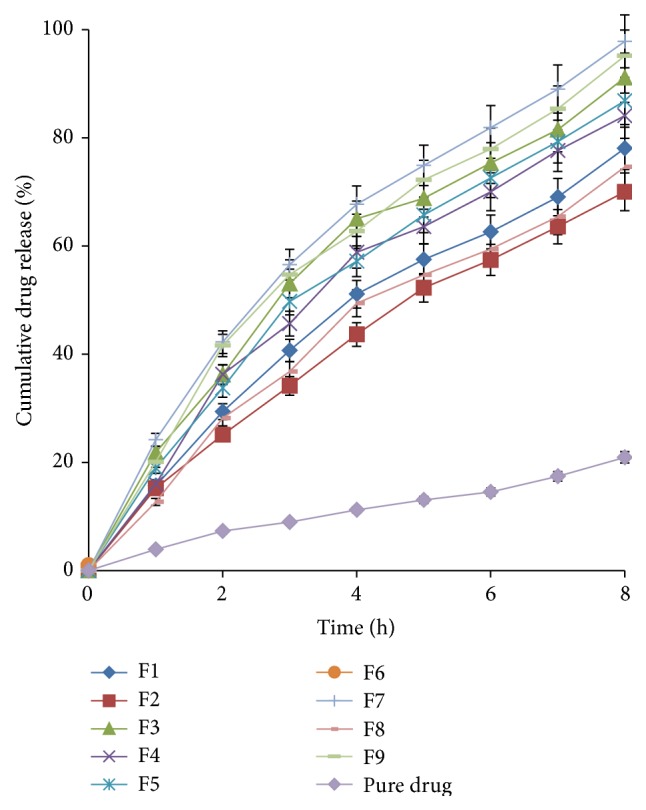
*In vitro* release profiles of acyclovir from cellulose acetate AMCs and pure drug in phosphate buffer, pH 4.5.

The drug release pattern of F7 was compared with the release profile of the extra design checkpoint formulation, F9. The % CDR of 95.20 ± 0.75% and a release profile comparable to F7 proved the feasibility of the formulation design. The release profile of the optimized formulation (F7) was also compared with pure drug (PD). Comparative* in vitro* drug release profile of F7 and PD (Figure 3) displayed superior sustenance of drug release from the formulation F7 over the drug packed in AMC in pure state (20.94%) proving that osmotically active formulation has better performance characteristics. The developed formulation has the potential to offer patient compliance by reducing the frequency of administration.

### 3.4. Kinetics

The* in vitro* release profiles of F1–F8 were modeled and the results showed that the best fit model for most of formulations was the zero-order model. F7 identified as optimized formulation displayed controlled drug release owing to the fact that coefficient of determination (*r* ^2^) was 0.9898 (Table 5). Cellulose acetate membranes are reported to generate semipermeable membranes of controlled porosity that have been utilized for osmotic pump-based controlled release systems [26]. The results of the present work are closely correlated with those cited in the literature.

### 3.5. *In Vitro* Buoyancy

Visual monitoring of the formulations during* in vitro* drug release studies in phosphate buffer, pH 4.5, exhibited buoyancy till 8 h. The capsule shell was composed of cellulose acetate 398-10 that has a density of about 0.4 g/cm^3^ [27], which is much lower than the density of gastric fluid (1.004 g/cm^3^), and hence assisted floating of the AMC. All the remaining excipients were water-soluble; therefore the floating ability is corelatable to cellulose acetate 398-10. To confirm the buoyant characteristics of the capsule, the true density of the optimized F7 was found to be 0.398 g/cm^3^ when determined by liquid displacement method using ethanol 95% v/v as the displacement liquid. Accordingly, the AMCs floated in the release media for 8 h, thus affirming the use of AMCs as gastroretentive system for a poorly soluble drug.

### 3.6. Statistical Analysis

**Figure 4:**
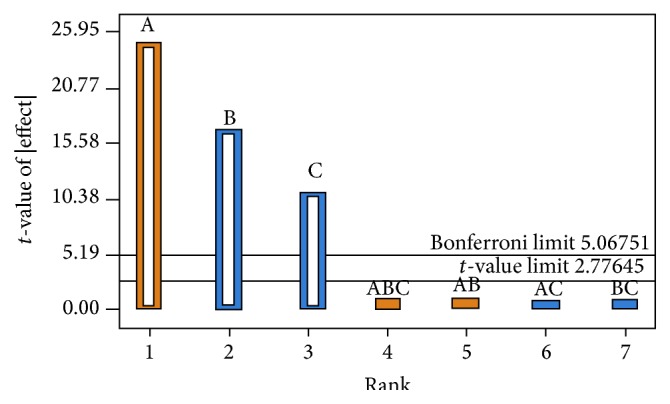
Pareto chart depicting significant coefficients above the Bonferroni line for the response cumulative drug release.

Statistical analysis was carried out using Design Expert software version 9 (Stat-Ease, Inc., Minneapolis, USA). The effect of independent variables can be described by polynomial equation generated by Pareto chart (Figure 4). Bonferroni limit line (*t*-value of effect = 5.06751) and *t*-limit line (*t*-value of effect = 2.77645) were generated by the software. The coefficients having *t*-value between Bonferroni lines were called certainly significant coefficient and, the *t*-value of effect between Bonferroni line and *t*-limit line was called likely to be significant coefficient, while the *t*-values below the *t*-limit line were called statistically insignificant coefficient [13] and were removed from the analysis.

The significant response polynomial equation generated for response parameter (CDR) was

(2) $$\begin{array}{c} \text{\% }{\text{CDR}}_{8\;h}=95.87-3.45\;\left(\;A\;\right)+2.46\;\left(\;B\;\right)+0.74\;\left(\;C\;\right). \end{array}$$

**Figure 5:**
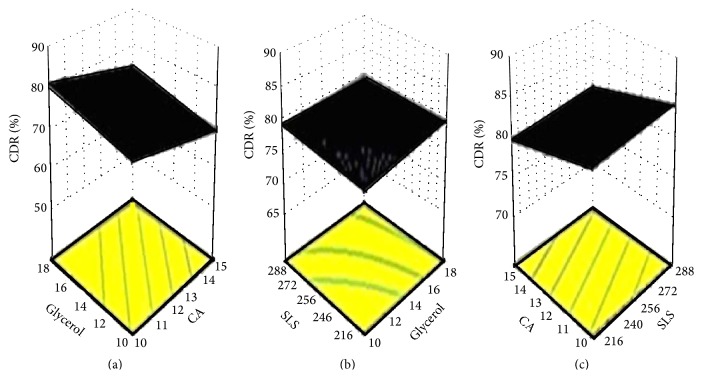
3D response surface plots depicting the simultaneous effect of independent variables on the response (%  CDR_8 h_).

The equation suggests the negative impact of cellulose acetate (A) on the* in vitro* drug release whereas glycerol (B) and sodium lauryl sulphate (C) contributed positively to the release of acyclovir from AMCs. In order to analyze the effect of varying levels of independent variables the response surface plots were analyzed. The 3D surface response graphs depict the simultaneous influence of independent variables on the dependent variables (Figure 5). On simultaneous increase of level of glycerol and cellulose acetate, the % CDR was increased (Figure 5(a)) whereas simultaneous increase in levels of SLS and glycerol did not influence the % CDR (Figure 5(b)). Whereas a concurrent increase in the levels of cellulose acetate and SLS influenced the CDR characteristically (Figure 5(c)). The positive effect of SLS was counteracted with cellulose acetate levels.

### 3.7. Interaction between Independent Factors

**Figure 6:**
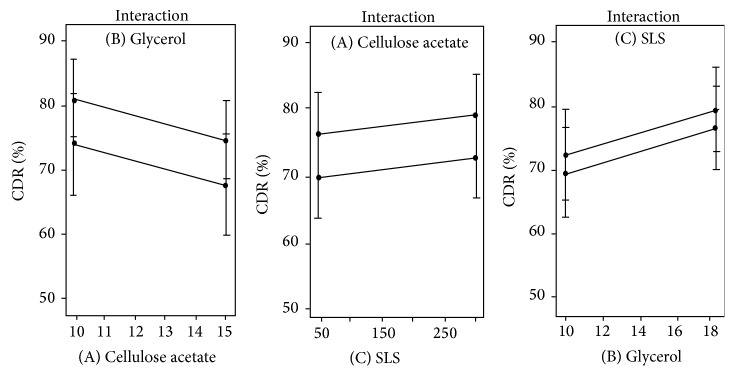
Interaction plots of independent variables.

The possible interactions between independent variables AB, AC, and BC on the response parameter were studied by interaction plots (Figure 6). Graphically, interaction can be visualized by the lack of parallelism in the lines. In our case the parallel lines indicated no interaction between the two variables which indicated independency of variables. Thus the design has maximum efficiency in estimating effects of variables on drug release [28].

### 3.8. Selection of Optimized Formulation and Validation of Experimental Design

On the basis of the % CDR of 97.88% ± 0.77, *r* ^2^ = 0.9898, and maximum desirability of 0.871 F7 was selected as the optimized formulation. The experimental design was validated by preparing an extra design checkpoint formulation F9 (Table 1) and evaluated. The close resemblance between predicted and experimental value ascertained the validity of experimental design. The predicted value of %CDR_8 h_ was deduced as 95.87% and the experimental value was 95.20%. Low value (0.70%) of percentage error between predicted and experimental values affirmed the prognostic ability of the design. The* in vitro* release profile of acyclovir from F7 was statistically compared with the theoretical formulation (extra design checkpoint). The statistical significance was tested at *p* < 0.05. The formulation F7 displayed high similarity factor (*f*2) of 74.11.

### 3.9. Effect of Variables on Drug Release

#### 3.9.1. Effect of Varying Speed

**Figure 7:**
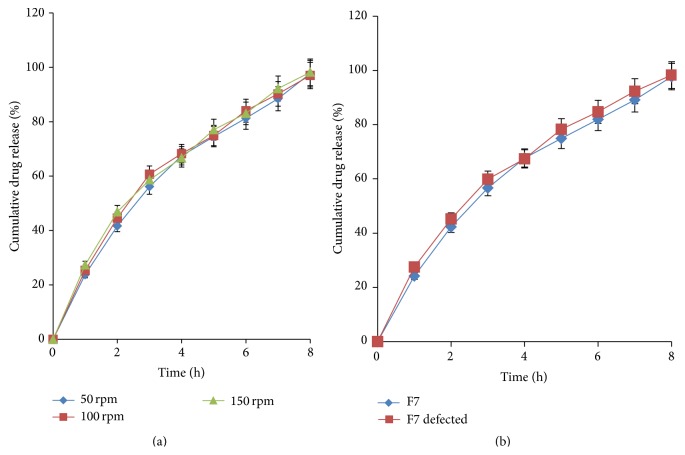
(a) Effect of agitational intensity on* in vitro* release and (b) effect of intentional defect on* in vitro* release of acyclovir from cellulose acetate AMC.

The* in vitro* release profiles at three different speeds 50, 100, and 150 rpm were compared using one-way ANOVA (Figure 7(a)). Rotation rates outside 25 to 150 rpm limits are usually inappropriate because of the inconsistency of hydrodynamics below 25 rpm and because of turbulence above 150 rpm [29]. The calculated *F*-value (0.02052) was found to be less than tabulated value (1.029), thus suggesting that the variation in agitational intensity did not affect the release profile of the drug from the AMC (Figure 7(a)). This effect describes the fact that the* in vitro* release from the AMCs is independent of the hydrodynamic conditions of the body because of semipermeable nature of the rate controlling membrane and design of delivery orifice used in osmotic systems. Thus, it can be stated that drug release from F7 was due to the controlled entry of dissolution medium across the cellulose acetate barrier and not due to turbulence in dissolution medium.

#### 3.9.2. Effect of Intentional Defect

To establish that the defects in the asymmetric membrane on the release kinetics will not influence the release, intentional defect (pore of 2 mm dia) was introduced in capsular membrane and* in vitro* release studies were accomplished on F7 (Figure 7(b)). The release profile of intentionally defected F7 was compared with optimized F7 using one-way ANOVA. The calculated *F*-value (0.038) and *t*-value (0.5905) were found to be less than tabled *F*-value (4.60) and *t*-value (2.36). These results demonstrate that release kinetics from AMCs was independent of defects in the membrane, a unique property of osmotic devices [30].

#### 3.9.3. Effect of Varying Osmotic Pressure

**Figure 8:**
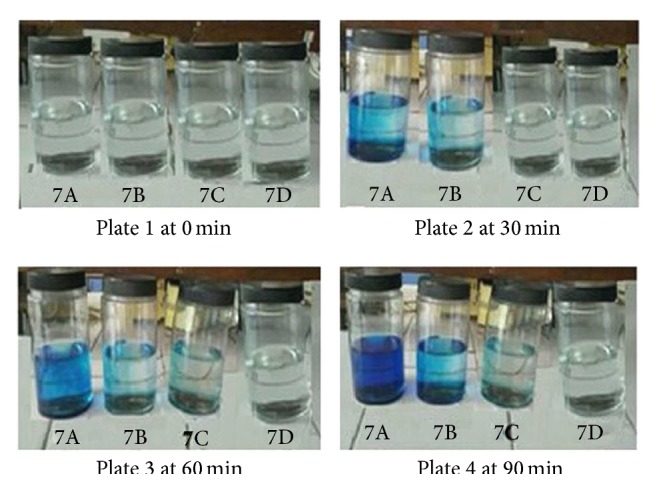
Images depicting the effect of varying osmotic conditions of dye release from AMC, plate 1 at 0 min, plate 2 after 30 min, plate 3 after 60 min, and plate 4 after 90 min.

The capsules were visually studied for release of dye which indicates its osmotic expulsion from core of capsule.  Figure 8 shows four plates, 1, 2, 3, and 4, showing release at 0 min, 30 min, 60 min, and 90 min, respectively. F7A showed solubility dependent release of methylene blue from the AMCs that intensified with time (plates 1–4). F7B comprising both the dye and the osmotic agent in the AMC showed gradual release suggesting an osmotic pressure gradient across the capsule membrane.

**Figure 9:**
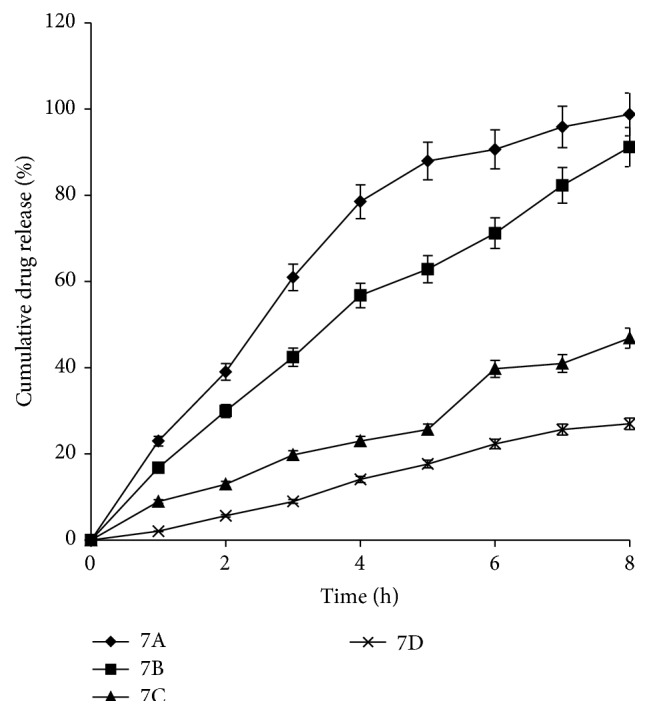
7A: without osmogen inside and outside the capsule (zero osmotic gradient); 7B: 290 mg SLS inside the capsule and 0 mg outside the capsule (perfect osmotic gradient); 7C: 290 mg SLS inside and 145 mg outside the capsule (hypoosmotic); 7D: 290 mg SLS inside and 430 mg in the media (hyperosmotic).

The dye release was initiated after 30 min of immersion of the AMC in the solution due to the gradual build-up of osmotic gradient that facilitated the release of the dye in a sustained manner. F7C and F7D were kept in hypoosmotic and hyperosmotic conditions, respectively, to demonstrate that the principle mechanism of drug delivery was the property of osmogen incorporated into the formulation. As the osmotic gradient was reduced, delayed methylene blue release was seen in F7C. No color was observed for F7D in all the plates indicating zero release of methylene blue from the AMC under hyperosmotic conditions conforming to osmotic gradient as essential requirement for drug release. This confirmed that the mechanism of drug release from the developed AMCs was solely dependent on the osmotic pressure gradient across the AMCs and the surrounding environment drug release is inversely proportional to the osmotic pressure. Quantitative assessment of varying the osmotic pressure on the drug release was also analyzed.  Figure 9 displays that the drug release is inversely proportional to the osmotic pressure. It is evident that the drug release decreased as the osmotic pressure of the release media increased. The release profiles, when compared using one-way ANOVA, led to a calculated *F*-value of 2.91, which was more than the tabulated *F*-value of 2.78. This confirmed that the mechanism of drug release from the developed AMC was solely dependent on the osmotic pressure gradient across the asymmetric membrane.

### 3.10. Scanning Electron Microscopy (SEM)

**Figure 10:**
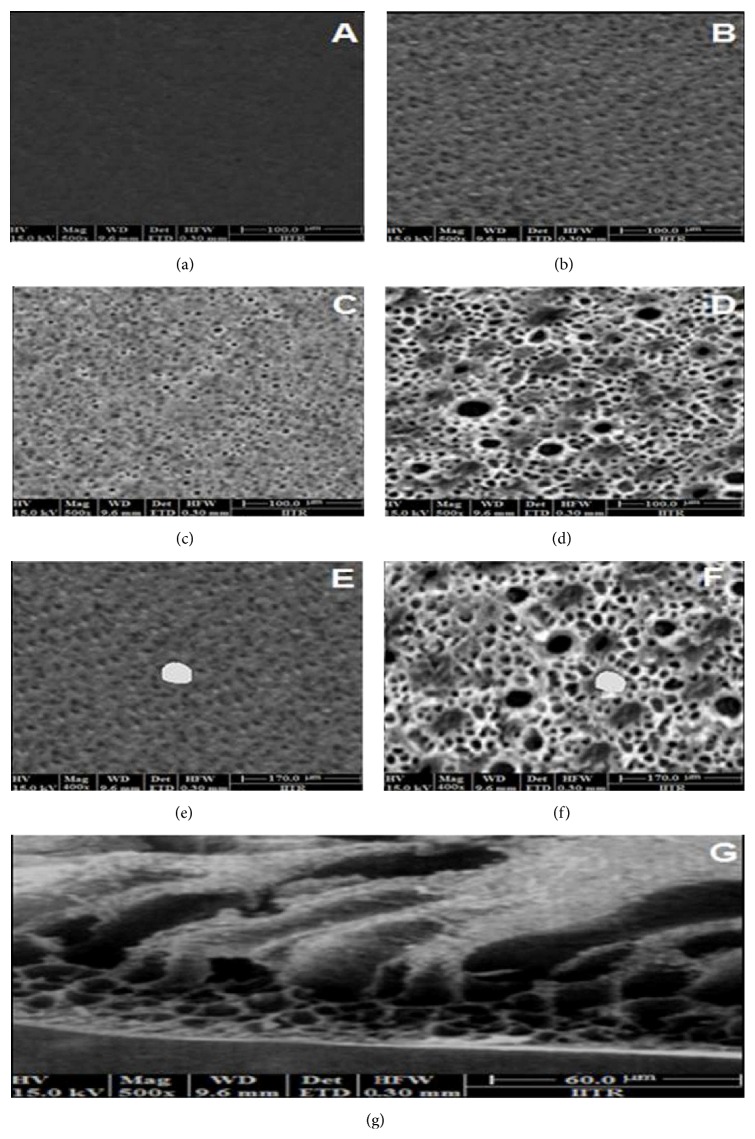
SEM of asymmetric membrane depicting (a) outer dense region and (b) inner porous region before drug release (original magnification at 500x), (c) outer dense region and (d) inner porous region after drug release (original magnification at 500x), (e) inner surface with intentional defect before dissolution and (f) inner surface with intentional defect after dissolution (400x), and (g) transverse section (500x).

The SEM images of asymmetric membrane obtained before dissolution clearly indicated an outer, dense, nonporous, and smooth membranous structure of cellulose acetate (Figure 10(a)) at 500x, while the inner surface was porous and rough (Figure 10(b)). Numerous pores on the outer surface of membrane (Figure 10(c)) and large pores (0.73 *μ*m in diameter) in the inner layer of membrane (Figure 10(d)) were clearly seen. The pore enlargement is attributed to the dissolution of glycerol into the aqueous media during drug release. With* in vitro* release study the permselectivity of the outer membrane also increased. The original concept was to form an asymmetric membrane film consisting of a thick porous region to provide mechanical support and a thin dense region to provide permselectivity. Thus, a significant factor in determining the permeability of a given membrane is the porous nature of the membrane. The membrane structure is controlled by both the formulation and processing variables [23]. Intentionally defected asymmetric membrane showed a 2 mm hole (Figure 10(e)) which remained almost of the same size even after 8 h of dissolution (Figure 10(f)). Transversally sectioned micrograph of the capsule shell (Figure 10(g)) clearly shows the asymmetric nature of membrane with an outer thin dense region supported by an inner porous substructure with deep micropores.

### 3.11. Stability

**Figure 11:**
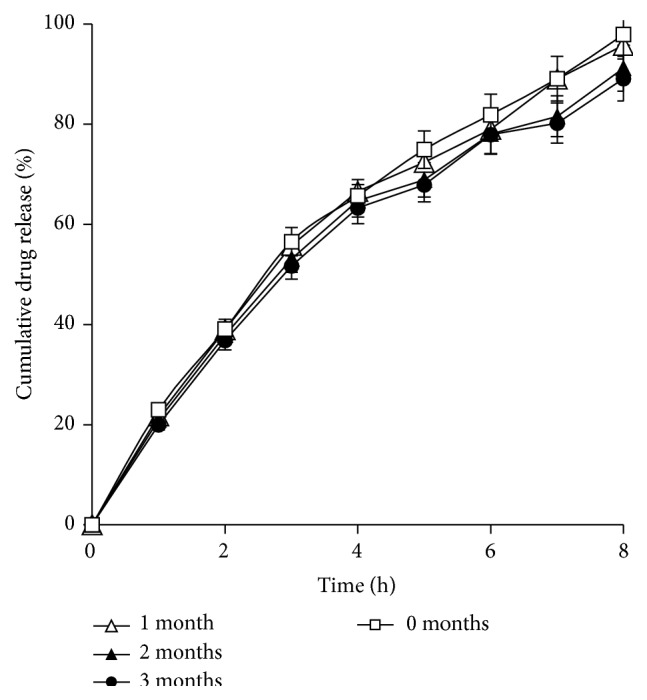
*In vitro* drug release profiles of fresh and aged F7 formulation.

The result showed that the formulation was stable at 40 ± 2.0°C/75 ± 5% RH. No change in the capsule texture and color was seen. The percent drug content profile showed comparable results, thus suggesting that there was no stability issue for AMCs (Table 6). The* in vitro* drug release in phosphate buffer, pH 4.5, showed insignificant changes in the release profiles (*p* > 0.05), *f*2 > 85 confirming stability in the release of drug from AMCs even after storage (Figure 11).

## 4. Conclusion

The cellulose acetate AMCs of acyclovir were successfully developed that showed osmotically driven controlled release of the drug, independent of the agitational intensity and intentional defect on the membrane. Osmotic gradient was the primary mechanism for the release of poorly water-soluble drug acyclovir from the buoyant AMC. The levels of cellulose acetate influenced the performance characteristics of the AMCs but did not influence the buoyancy. Designing a system that can be retained at the site of absorption may resolve the poor permeability issues but this needs to be proved by experimental studies* in vivo*. However, the generated system has the potential to improve patient compliance by sustenance of drug release and to improve bioavailability by zero-order release of the drug.

## Figures and Tables

**Table 1:** Actual and coded values of independent variables used for fabrication of AMCs of acyclovir in 2^3^ full factorial design.

Formulation code	Cellulose acetate 398-10 (% w/v) (A)	Glycerol (% v/v) (B)	Sodium lauryl sulphate (mg) (C)	Dependent variable
F1	10 (−1)	10 (−1)	215 (−1)	% CDR_8 h_^b^ (Y1)
F2	15 (+1)	10 (−1)	215 (−1)	
F3	10 (−1)	18 (+1)	215 (−1)	
F4	15 (+1)	18 (+1)	215 (−1)	
F5	10 (−1)	10 (−1)	290 (+1)	
F6	15 (+1)	10 (−1)	290 (+1)	
F7	10 (−1)	18 (+1)	290 (+1)	
F8	15 (+1)	18 (+1)	290 (+1)	
F9^a^	12	(14)	(250)	

^a^Extra design checkpoint; ^b^cumulative drug release.

**Table 2:** Various osmotic conditions used for studying the effect of osmotic pressure on drug release from asymmetric membrane capsule of acyclovir.

AMC code	SLS in AMC (mg)	SLS outside AMC (mg)	Osmotic condition	Result
F7A	0	0	Absent	Dye release intensified with time
F7B	290	0	Perfect osmotic gradient	Controlled release
F7C	290	145	Hypoosmotic	Slight release
F7D	290	430	Hyperosmotic	No release

**Table 3:** Physical characterization of asymmetric membrane capsule (AMC) as compared to conventional hard gelatin capsule (HGC).

Type of capsule	Appearance	Dimensions (mm)				
		Cap		Body		Lock length
		Length	Diameter	Length	Diameter	
HGC	Transparent	10.88 ± 0.10	7.52 ± 0.02	18.60 ± 0.12	7.24 ± 0.04	21.32 ± 0.09
AMC	Opaque	10.89 ± 0.16	7.74 ± 0.01	18.74 ± 0.08	7.31 ± 0.09	21.58 ± 0.30

**Table 4:** Percent drug content, weight uniformity, and cumulative drug release data of the asymmetric membrane capsules of acyclovir.

Formulation code	Weight^#^ (mg) ± S.D.	Drug content^##^ (%) ± S.D.	CDR_8 h_ (%) ± S.D.
F1	697.60 ± 1.18	97.74 ± 0.55	70.07 ± 1.93
F2	697.80 ± 1.05	98.21 ± 0.60	70.04 ± 1.36
F3	697.95 ± 0.99	98.36 ± 0.54	91.16 ± 0.30
F4	698.40 ± 0.75	92.61 ± 1.44	84.11 ± 0.95
F5	769.10 ± 0.91	94.63 ± 1.08	80.59 ± 1.48
F6	768.75 ± 1.06	93.23 ± 1.33	74.63 ± 0.46
F7	769.35 ± 0.74	99.45 ± 0.37	97.88 ± 0.77
F8	769.00 ± 1.02	96.65 ± 1.51	86.86 ± 1.25
F9^*∗*^	732.95 ± 1.09	97.12 ± 1.08	95.20 ± 0.75

^#^Average of 20 determinations; ^##^average of five determinations.

^*∗*^Extra design check point.

**Table 5:** Kinetic modelling of *in vitro *release data of acyclovir from asymmetric membrane capsules.

Formulation code	*r* ^2^			
	Zero-order	First-order	Higuchi	Hixson-Crowell
F1	0.9768	0.5417	0.9667	0.6886
F2	0.9784	0.5501	0.9527	0.6983
F3	0.9845	0.5072	0.9408	0.6520
F4	0.9782	0.5350	0.9507	0.6761
F5	0.9780	0.5407	0.9594	0.6850
F6	0.9719	0.5499	0.9453	0.6853
F7	0.9898	0.4997	0.9448	0.6465
F8	0.9818	0.5270	0.9581	0.6741
F9^*∗*^	0.9880	0.5141	0.9476	0.6606

^*∗*^Extra design check point.

**Table 6:** Stability data cellulose acetate AMCs of acyclovir for 3 months.

Parameter	Time interval (months)			
	0	1	2	3
Appearance	White	White	White	White
Surface	Smooth	Smooth	Smooth	Smooth
Drug content (%)	99.45	99.14	98.83	98.52

## References

[1] Herbig S. M., Cardinal J. R., Korsmeyer R. W., Smith K. L. (1995). Asymmetric-membrane tablet coatings for osmotic drug delivery. *Journal of Controlled Release*.

[2] Wang C.-Y., Ho H.-O., Lin L.-H., Lin Y.-K., Sheu M.-T. (2005). Asymmetric membrane capsules for delivery of poorly water-soluble drugs by osmotic effects. *International Journal of Pharmaceutics*.

[3] Thombre A. G., DeNoto A. R., Gibbes D. C. (1999). Delivery of glipizide from asymmetric membrane capsules using encapsulated excipients. *Journal of Controlled Release*.

[4] Chauhan M. S., Kumar A., Pathak K. (2012). Osmotically regulated floating asymmetric membrane capsule for controlled site-specific delivery of ranitidine hydrochloride: optimization by central composite design. *AAPS PharmSciTech*.

[5] Choudhury P. K., Ranawat M. S., Pillai M. K., Chauhan C. S. (2007). Asymmetric membrane capsule for osmotic delivery of flurbiprofen. *Acta Pharmaceutica*.

[6] Garg A., Gupta M., Bhargava H. N. (2007). Effect of formulation parameters on the release characteristics of propranolol from asymmetric membrane coated tablets. *European Journal of Pharmaceutics and Biopharmaceutics*.

[7] Waterman K. C., Goeken G. S., Konagurthu S. (2011). Osmotic capsules: a universal oral, controlled-release drug delivery dosage form. *Journal of Controlled Release*.

[8] Dhaliwal S., Jain S., Singh H. P., Tiwary A. K. (2008). Osmotic capsules: a universal oral, controlled-release drug delivery dosage form. *The AAPS Journal*.

[9] Kristl A., Srčič S., Vrečer F., Šuštar B., Vojnovic D. (1996). Polymorphism and pseudopolymorphism: influencing the dissolution properties of the guanine derivative acyclovir. *International Journal of Pharmaceutics*.

[10] (2004). *AHFS Drug Information*.

[11] Fletcher C., Bean B. (1985). Evaluation of oral acyclovir therapy. *Drug Intelligence and Clinical Pharmacy*.

[12] Rawlins E. A. (2004). *Bentley's Textbook of Pharmaceutics*.

[13] Bolton S. (1990). *Pharmaceutical Statistics: Practical and Clinical Application*.

[14] Dev R., Kumar A., Pathak K. (2012). Solubility-modulated asymmetric membrane tablets of triprolidine hydrochloride: statistical optimization and evaluation. *AAPS PharmSciTech*.

[15] (2007). *Indian Pharmacopoeia, Government of India, Ministry of Health & Family Welfare*.

[16] http://www.ich.org/products/guidelines/quality/quality-single/article/stability-testing-of-new-drug-substances-and-products.html.

[17] Kristl A. (1999). Estimation of aqueous solubility for some guanine derivatives using partition coefficient and melting temperature. *Journal of Pharmaceutical Sciences*.

[18] Luengo J., Aránguiz T., Sepúlveda J., Hernández L., Von Plessing C. (2002). Preliminary pharmacokinetic study of different preparations of acyclovir with *β*-cyclodextrin. *Journal of Pharmaceutical Sciences*.

[19] Kumar A., Philip A. K., Pathak K. (2011). Asymmetric membrane capsules of phenylephrine hydrochloride: an osmotically controlled drug delivery system. *Current Drug Delivery*.

[20] Keraliya R. A., Patel C., Patel P. (2012). Osmotic drug delivery system as a part of modified release dosage form. *ISRN Pharmaceutics*.

[21] Budavari S. (1996). *The Merck Index: An Encyclopedia of Chemicals, Drugs and Biological*.

[22] Liu L., Khang G., Rhee J. M., Lee H. B. (1999). Preparation and characterization of cellulose acetate membrane for monolithic osmotic tablet. *Korea Polymer Journal*.

[23] Philip A. K., Pathak K. (2006). Osmotic flow through asymmetric membrane: a means for controlled delivery of drugs with varying solubility. *AAPS PharmSciTech*.

[24] Philip A. K., Pathak K. (2007). In situ-formed asymmetric membrane capsule for osmotic release of poorly water-soluble drug. *PDA Journal of Pharmaceutical Science and Technology*.

[25] Philip A. K., Pathak K., Shakya P. (2008). Asymmetric membrane in membrane capsules: a means for achieving delayed and osmotic flow of cefadroxil. *European Journal of Pharmaceutics and Biopharmaceutics*.

[26] Makhija S. N., Vavia P. R. (2003). Controlled porosity osmotic pump-based controlled release systems of pseudoephedrine: I. Cellulose acetate as a semipermeable membrane. *Journal of Controlled Release*.

[27] Rowe R. C., Shesky P. J., Owens S. C. (2006). *Handbook of Pharmaceutical Excipients*.

[28] Shah M. K., Pathak K. (2010). Development and statistical optimization of solid lipid nanoparticles of simvastatin by using 2^3^ full-factorial design. *AAPS PharmSciTech*.

[29] (2004). *United States Pharmacopoeia*.

[30] Donald L. W. (2000). *Handbook of Pharmaceutical Controlled Release Technology*.

